# Hereditary hemorrhagic telangiectasia patient presenting with brain abscess due to silent pulmonary arteriovenous malformation

**DOI:** 10.11604/pamj.2016.25.145.11010

**Published:** 2016-11-11

**Authors:** Marios Themistocleous, Dimitrios Giakoumettis, Andreas Mitsios, Christos Anagnostopoulos, Aristoteles Kalyvas, Christos Koutsarnakis

**Affiliations:** 1 Department of Neurosurgery, Children’s Hospital “Agia Sofia”, Athens, Greece; 2 Department of Neurosurgery, University of Athens Medical School, "Evangelismos" General Hospital, Athens, Greece

**Keywords:** Hereditary hemorrhagic teleangiectasia, brain abscess, arteriovenous malformation

## Abstract

Hereditary hemorrhagic telangiectasia is a rare autosomal dominant inherited disease that is usually complicated by visceral vascular malformations. Patients harboring such malformations are at increased risk of brain abscess formation, which despite advances in diagnostic and surgical methods remains a life threatening medical emergency with high mortality and morbidity rates. In the present report we describe a case of cerebral abscess due to silent pulmonary arteriovenous malformation (AVM) in a young patient previously undiagnosed for hereditary hemorrhagic telangiectasia syndrome (HHT).

## Introduction

Hereditary hemorrhagic telangiectasia (HHT) also known as Osler-Weber-Rendu syndrome is a rare systemic angiodysplasia inherited as an autosomal dominant disorder. Approximately 1% of patients with HHT are expected to develop a brain abscess [1, 2]. However, it has to be noted that this percentage may rise up to 10% in HHT patients affected by pulmonary arteriovenous malformations (PAVM) [1]. This report describes a case of cerebral abscess due to silent pulmonary arteriovenous malformation (AVM) in a young patient previously undiagnosed for hereditary hemorrhagic telangiectasia syndrome (HHT).

## Patient and observation

**Figure 1:**
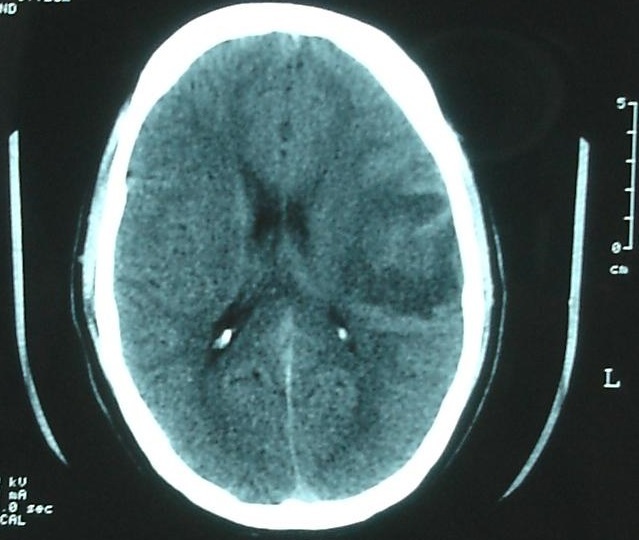
CT image showing a heterogeneous mass with surrounding brain edema in the left temporal region

**Figure 2:**
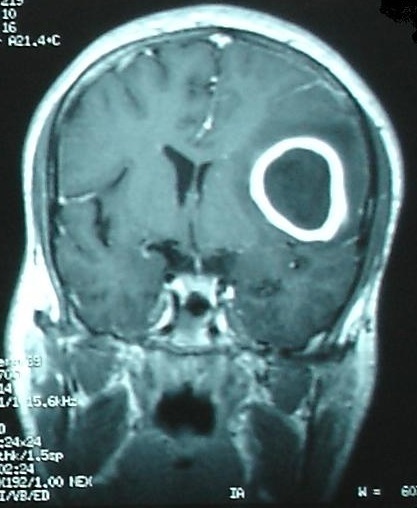
A coronal T1-weighted MRI image with Gadolinium contrast demonstrating peripheral enhancement of the lesion in the left hemispher

**Figure 3:**
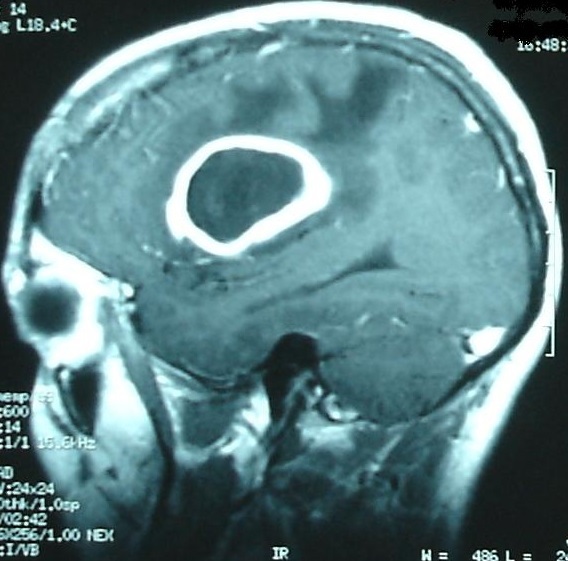
A sagittal T1-weighted MRI image with Gadolinium contrast illustrating the lesion with peripheral enhancement surrounded by edema

**Figure 4:**
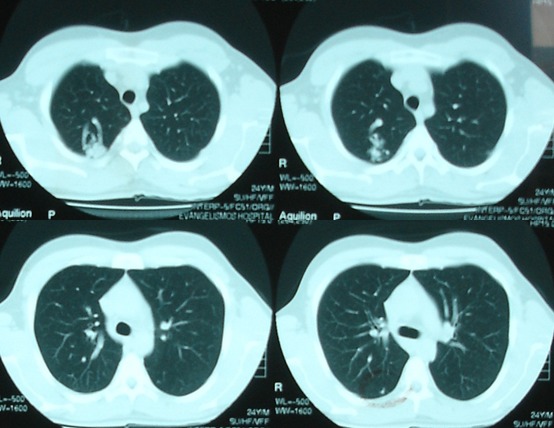
Chest CT scan showed AVM in the right upper lobe

A 24-year-old male presented to the Emergency Department of Evangelismos Hospital, due to generalized tonic clonic seizures accompanied by fever, increasing right hemiparesis and motor dysphasia. Brain CT and MRI scans (Figure 1, Figure 2, Figure 3), revealed a left frontal cystic mass with surrounding edema and ring enhancement, following contrast administration. The patient was then admitted to our department and treatment with anticonvulsants and corticosteroids was promptly initiated. On close clinical inspection, the presence of cutaneous and mucosal telangiectasias along with a positive medical history of recurrent epistaxis led to an initial diagnosis of HHT indicating that the detected mass was an abscess, until proven otherwise. Urgent aspiration through a burr hole was performed and 20 ml of pus were removed leading to the marked improvement of the patient's neurological condition. A chest CT scan (Figure 4) showed the presence of pulmonary arteriovenous malformations (PAVM) in both lungs, thus confirming a potential source of the brain abscess. Corticosteroids were tapered and ultimately discontinued. Pus cultures obtained intraoperatively were negative and the patient was treated for 4 weeks with intravenous administration of vancomycin (500mg/8 hours) and ceftriaxone (1gr/12 hours). Hemiparesis and speech difficulties gradually improved with the aid of speech and physical therapy and a brain MRI obtained 6 weeks after, showed complete resolution of the abscess. Following discharge, the patient underwent a pulmonary angiography and embolotherapy.

## Discussion

Hereditary hemorrhagic telangiectasia (HHT) also known as Osler-Weber-Rendu syndrome is a rare systemic angiodysplasia inherited as an autosomal dominant disorder. Four diagnostic criteria for HHT have been proposed i) epistaxis ii) telangiectasia on face, fingertips and nasal/oral mucosa iii) familial history of HHT and iv) visceral vascular anomalies including hepatic, pulmonary, spinal and cerebral arteriovenous malformations [3]. Diagnosis of HHT is certain if three out of four criteria are satisfied, suspected if two out of four are fulfilled and unlikely if only one criterion is met. Approximately 1% of patients with HHT are expected to develop a brain abscess [1, 2]. However, it has to be noted that this percentage may rise up to 10% in HHT patients affected by pulmonary arteriovenous malformations (PAVM) [4]. PAVMs that are characterized by pathological connections between an afferent artery and one or more efferent veins without an interposed capillary bed, provide an extra cardiac right to left shunt as well as a bypass from the filtering effect of lung capillaries. Given time, thromboemboli and septic microemboli arising in the pulmonary circulation may evoke cerebrovascular disorders one of which is the formation of a brain abscess. The close interconnection between HHT and PAVMs is supported by previous findings indicating that half of the patients harboring PAVMs suffer from HHT whereas 15-33% of HHT patients have PAVMs [5]. Typically PAVMs remain asymptomatic and neurological complications, the most serious of which is brain abscess formation, can be their first clinical manifestation [2, 6]. Treatment of a brain abscess in a HHT patient with PAVM requires the initial aspiration or excision of the abscess followed by embolization of the PAVM. Previous findings indicating significantly higher mortality rates (approximately 40%) in HHT patients harboring brain abscesses compared to brain abscess patients without HTT (10-15%), highlight the critical importance of prompt diagnosis and treatment in the former patient group [7–9].

## Conclusion

Our case illustrates that a brain abscess may be the first clinical manifestation of a nearly asymptomatic HHT patient. Furthermore patients with a history suggestive of HHT should be screened for the presence of visceral vascular malformations as they are at increased risk of brain abscess formation. In conclusion, it should be clearly pointed out not only to neurologists and neurosurgeons but to clinicians in general, that patients with signs suggestive of HHT and new onset of neurological symptoms, should be deemed to harbor a brain abscess until proven otherwise. This aggressive approach is mandated by the high prevalence and mortality associated with a brain abscess in this group of patients.

## References

[1] Press OW, Ramsey PG (1984). Central nervous system infections associated with hereditary hemorrhagic telangiectasia. Am J Med..

[2] Hall WA (1994). Hereditary hemorrhagic telangiectasia (Osler-Weber-Rendu Disease) presenting with polymicrobial brain abscess. J Neurosurg..

[3] Shovlin CL, Hughes JM, Tuddenham EG, Temperley I, Perembelon YF, Scott J, Seidman CE, Seidman JG (1994). A gene for hereditary haemorhagic telangiectasia maps to chromosome 9q. Nat Genet..

[4] Dong SL, Reynolds SF, Steiner IP (2001). Brain abscess in patients with hereditary hemorrhagictelangiectasia: Case report and literature review. Emerg Med..

[5] Greenberg MS, Greenberg MS (2006). Cerebral abscess. Handbook of Neurosurgery..

[6] Momma F, Ohara S, Ohyama T, Moto A, Okada H, Harada H (1990). Brain abscess associated with congenital pulmonary arteriovenous fistula. Surg Neurol..

[7] Grigoriadis E, Gold WL (1997). Pyogenic brain abscess caused by streptococcus pneumoniae: case report and review. Clin Infect Dis..

[8] Seydoux CH, Francioli P (1992). Bacterial brain abscesses: factors influencing mortality and sequelae. Clin Infect Dis..

[9] Svanteson B, Nordstrom CH, Rausing A (1988). Non-traumatic brainabscess: epidemiology, clinical symptoms and therapeutic results. Acta Neurochir (Wien..

